# On a Ferruginous Preparation, Termed, Vulgarly, “Clinkers,” in Certain Diseases

**Published:** 1844-02

**Authors:** W. S. McMurtry

**Affiliations:** Benton, Mississippi; Louisville


### WESTERN JOURNAL

OF

### MEDICINE AND SURGERY.

---

FEBRUARY, 1844.

---

ART. I.—*On a Ferruginous preparation, termed, vulgarly, "Clinkers," in certain Diseases.* By W. S. McMurtry, M.D., of Benton, Miss. Being an Inaugural Dissertation, submitted to the President, Managers and Faculty of the Medical Institute of Louisville, for the degree of M.D. March, 1843.

In the march of investigation one fact of great value and interest has been ascertained and settled—that nature, by the operation of a few laws and a few agents, a few simple means, brings about an immense variety of important results. A knowledge of this fact is of the greatest importance to the philosopher, as it teaches him when new phenomena are discovered, not to seek for new laws and new agents as means of explanation, but if possible, to refer them to the combined operation of laws and agents already known. Thus we are frequently astonished after long research and investigation, to find at last that one or two simple truths explain the whole of a great number of phenomena which had been before entirely unintelligible. And still more are we astonished that these simple principles, which appear almost self-evident, should have been so long unknown. When a principle is once known it is difficult to say to what results it may lead.

Thus, the investigation of the cause of the contractions in the muscles of the leg of a frog, under peculiar circustances, has led to the discovery of Dynamic Electricity with all its wonderful effects. The same great principle is exhibited in numberless other cases. Among these, none is more admirable than the great variety of important purposes to which the various forms of Iron may be applied. I shall not stop to enumerate its application in the arts, but proceed at once to the subject of this paper—the remedial virtues of one of the forms of this metal. No class of remedies is more interesting on account of their active and varied properties than the chalybeates. This class furnishes tonics, laxatives, anthelmintics and astringents,—in all cases giving tone to the digestive apparatus and invigorating the whole system, as well as exhibiting the specific effects of each particular preparation.

The pharmaceutic preparations of iron are rapidly increasing in number. Modern chemistry has furnished us with several preparations of this metal, the properties of which are exceedingly interesting and valuable. I propose, however, to treat more particularly of a preparation which has been but lately introduced to our notice, and has received but little attention from the profession at large. It is not very strange that it should have been so long overlooked, for its external appearance is any thing but attractive, and one would be as apt to suspect almost any thing else of possessing valuable properties as this. I allude to what in the manufacturing districts of England is called "*Clinkers*," which are nothing more than the scoriæ produced in the smith's forge by the fusion of aluminous earth and other impurities with a small portion of iron. In what state the constituents of this compound exist is not known. Some have supposed from its appearance and external hardness that the metal becomes steel during the process of fusion with the clay; but this is uncertain, as no analysis of it has ever been made upon which reliance can be placed. Possibly it may contain some peculiar chemical compound upon which its valuable properties depend.

For the first notice of this article we are indebted to Dr. C. J. Edwards, of Bath, who, in the Provincial Medical and Surgical Journal, of February 5th, 1842, has given us some account of its properties and uses in the cure of cachectic diseases.

In the districts mentioned, the clinkers have been long used by "knowing old women," as a remedy in chlorotic disorders, and with such uniform success as to have obtained for the medicine the title of "specific."

The following is the mode of preparing it:—The bluest and heaviest pieces should be selected from the mass; that which is of a light color and less ponderous, being entirely inert and useless. Being carefully selected, it is to be finely levigated, which, by the way, is a work of great difficulty, owing to its extreme hardness and metalloid character. Any quantity of this powder may be mixed with a sufficient amount of molasses to form a stiff paste—to every eight ounces of this mass, half an ounce of magnesia and an equal quantity of ginger must be added. The mass thus formed is any thing but inviting to the eye, but its appearance may be improved by using honey instead of molasses, and adding a small quantity of peroxide of iron to the compound. The *modus operandi* of the magnesia and ginger is sufficiently evident. It may be well to observe, however, that unless the ginger, or some other stomachic, be combined with it, griping pains in the bowels are very apt to be produced by its use.

The directions for administering it are quite as unique as the formula for its preparation. It must be taken twice a day—morning and night—for three successive days, and then omitted for a like period; and so continued until the course that has been decided upon has been finished. The dose is a tea-spoonful. Absurd as these directions seem, they are not without their value; for experience has proved that constitutional irritation supervenes, unless some decided interval is allowed, at stated periods, during a course of this remedy.

Dr. Edwards first tested its powers in a number of patients in whom the exsanguineous state of the skin, and wasting of the muscular fibre, were indicative of the morbid manner in which the functions of the stomach, alimentary canal, and uterus, were carried on. Within a month from commencing the use of the clinkers a striking improvement took place in the appearance of the patients, and before the expiration of two months, every unfavorable symptom disappeared. One case in particular is well worthy of especial notice, on account of the scrofulous condition of the submaxillary glands, and the ulcerated state in which one of them had been for several years; this healed during the use of the new medicine.

"I may observe, en passant," says Dr. E., "that previous to the trial of the clinkers, this young woman had been under professional treatment for some time without deriving any benefit from it. The pulse was weak and nearly 100; catamenia irregular in appearance, variable in quantity, and unnatural in composition; tongue foul, and what perhaps may be termed hysterical hypochondriasis existed to a great degree. A gentle cathartic, of infusion of senna, with tartrate of potash, was given for a few successive days before the clinkers were commenced. She had not taken the new medicine six weeks when most of the distressing symptoms disappeared, and her personal appearance became so improved, that her friends scarcely recognized in the ruddy girl before them, the pallid and unhealthy creature she once had been."

It appears greatly to benefit cases of simple indigestion, a few doses being capable of removing the most distressing symptoms. In that peculiar condition of the secretion from the bowels which is said to favor the formation or propagation of worms, it has proved advantageous in two ways—one by its mechanical action, the other by its tonic properties. This was an accidental discovery made during its trial in a case of leucorrhœa.

When the medicine is taken for the first time, a train of symptoms frequently supervenes which would induce a stranger to its effects to regard it as a dangerous compound. A great weight is felt in the epigastric region, accompanied by a burning sensation in the stomach; sensations of sickness, followed by those of fainting, come on; these are soon relieved by eructations of flatus. Some complain of pains in the limbs, particularly in the joints, others of tightness across the forehead, with giddiness, while all are troubled with heat, dryness of the mouth, and great thirst. At the second dose these symptoms are moderated, and the third is generally taken without any unpleasant feeling. After it has been persevered in for a short time, sensations of a different character arise; these are hunger, and a feeling of health and energy to which, perhaps, the patient has been a stranger for many years; then the complexion, if pale, commonly receives a ruddy tint, and the muscular fibre becomes firm and enlarges. After the first dose the fæces are like pitch, the urine generally pale and large in quantity; the bowels, if previously costive, become regular in their action; the pulse increases in volume, and the skin is pleasantly relaxed. To sum up its medicinal properties;—it may be said that the clinkers are tonic, stimulant, anthelmintic, and colorific, adapted generally to leucophlegmatic habits, and especially to female diseases, where a chlorotic condition of the constitution prevails.

My attention being called to this article by the high encomiums of Dr. E., I determined, during the last summer, to test its virtues. Not having many suitable cases in my own practice, I requested several of my medical friends to give it a trial; and as the result of these experiments I can say, that the clinkers promise to be one of the most valuable chalybeate preparations belonging to the Materia Medica, when its properties and uses shall have been thoroughly ascertained.

It seems to be particularly adapted to all cases where debility of the intestinal tube, and constipation of the bowels exist, on account of its laxative properties. In all cases where I have used it, or known it to be used, it has kept the bowels gently open, even where the most obstinate constipation had previously prevailed. But it may be used as a most valuable tonic in cases accompanied with diarrhœa, by combining it with some astringent medicine. In one case of indigestion accompanied by diarrhœa of long standing, produced by dissipation, it was used in combinanation with catechu, with marked benefit; so much so, that I have been written to for the prescription, since my arrival at this place. It appears to me that most of the unpleasant effects arising from its use, mentioned by Dr. Edwards, might be obviated by properly regulating the dose, and combining it with other articles to suit each particular case. At least, I have not witnessed the disagreeable effects which he mentions. Doubtless, as in all cases where powerful remedies are employed, a proper discrimination is required in its use.

It may be exhibited according to the directions already given, or it may be administered in doses of from 20 to 40 grains, in molasses, or syrup, or any other adhesive vehicle; and should be always combined with some stomachic if it be not contra-indicated.

I have used it in several cases where a laxative and tonic were required, and always with the happiest effects—the greatest objection to the article being the unpleasant, gritty taste which it leaves in the mouth, owing to the difficulty of reducing it to an impalpable powder.

The following cases were furnished me by my friend, Dr. Sharp, who made trial of the article at my suggestion:

Case I.—A girl, about 12 years of age, had been in bad health for two years, supervening upon a badly cured intermittent fever. Countenance sallow; eye-lids œdematous; lips very pale; tongue presented a flabby and exsanguineous appearance; abdomen very much swollen; little appetite; digestion bad; bowels seldom moved without cathartic medicine; feet and legs œdematous; pulse 120 and weak. She had a paroxysm of fever every 24 hours, preceded by a chill. This patient had been confined to the house for two years, during which time she had been treated by several physicians without benefit.

The pulverized clinkers were given to her in 20 grain doses, three times a day, in a table-spoonful of honey. This was continued about six weeks, when the rosy hue had returned to her cheeks and prolabia, the œdema and abdominal swelling had subsided; her appetite was good and bowels regular.

After this, the medicine was continued in 5 grain doses, three times a day, for three months, at the expiration of which time she was so far recovered as to be able to go to school.

Case II.—A negro woman, aged 35, had given birth to her last child 14 years previously, from which time she had been afflicted with leucorrhœa; discharge profuse, purulent and greenish; considerable pain in the lumbar region and uterus at times; just able to walk, except about her monthly periods, when she was confined to her bed; menses small in quantity; leucorrhœal discharge more profuse at these periods, and frequently so acrid as to excoriate the parts with which it came in contact; hysterical symptoms; headache, with occasional giddiness; pulse 90 and weak; such enlargement of the abdomen that she had the appearance of a woman in the latter months of pregnancy; countenance pallid; general debility; tongue pale, flabby, and loaded with a thick fur; appetite bad and capricious; sour stomach, with great desire for antacids; bowels constipated, seldom moved unless by a cathartic; sleep disturbed and not refreshing. Being a favorite servant, the skill of a great many physicians had been exhausted upon her, but without affording her any relief.

She was put upon the clinkers according to the original recipe, except the ginger and magnesia, which were omitted. For three weeks the symptoms were aggravated by the use of the remedy. The bowels were moved very freely, showing the necessity of intermitting its use; the discharge from the vagina was also increased, and more offensive. In a word, she thought herself getting much worse. After this she commenced improving rapidly, the enlargement of the abdomen subsiding, and all the other symptoms improving. In a very short time she was so far recovered as to become pregnant, which had not happened before, during the fourteen years of her illness.

In all probability, the unpleasant purging in this case would not have occurred if the medicine had been given at longer intervals, or in smaller doses.

Case III.—A lady, 26 years of age, had been afflicted with leucorrhœa for several years occasionally, but she had borne children in the mean time. For the last three months before I saw her, the discharge had been much increased. In addition to this, she was greatly harrassed with what she represented to be boils, about the size of a hickory-nut, located on the external labia and perineum—one crop of two or three would make their appearance, and as they declined be followed by another; she was very much debilitated by the irritation thus constantly kept up; bowels constipated; considerable distension of the abdomen; pulse weak and frequent; tongue flabby and loaded with a yellow fur; no appetite; acidity of the stomach; headache, with occasional giddiness and blindness; pain in the lumbar region, also in the right side, extending to the shoulder; urine high colored and scanty.

A tea-spoonful of the powdered clinkers in a little honey was ordered three times a day, with intermissions, as already mentioned. Cooling lotions were applied to the boils.

She commenced improving immediately, and in the course of a month was entirely cured.

Dr. Sharp informed me that he had used this remedy in several other cases, where a tonic was required, with marked benefit, and never with any unpleasant consequences; it always operating *gently* upon the bowels, except in the case of the negro woman, whose case has been detailed, in which instance it purged powerfully.

Louisville, March, 1843.

